# Developing an Agent-Based Drug Model to Investigate the Synergistic Effects of Drug Combinations

**DOI:** 10.3390/molecules22122209

**Published:** 2017-12-14

**Authors:** Hongjie Gao, Zuojing Yin, Zhiwei Cao, Le Zhang

**Affiliations:** 1 College of Computer and Information Science, Southwest University, Chongqing 400715, China; ghjbarry@126.com; 2 School of Life and Technology, Tongji University, Shanghai 200092, China; ZuoJing_Yin@tongji.edu.cn; 3 College of Computer Science, Sichuan University, Chengdu 610065, China

**Keywords:** agent-based model, parameter estimation, synergistic effect, drug combination, optimization

## Abstract

The growth and survival of cancer cells are greatly related to their surrounding microenvironment. To understand the regulation under the impact of anti-cancer drugs and their synergistic effects, we have developed a multiscale agent-based model that can investigate the synergistic effects of drug combinations with three innovations. First, it explores the synergistic effects of drug combinations in a huge dose combinational space at the cell line level. Second, it can simulate the interaction between cells and their microenvironment. Third, it employs both local and global optimization algorithms to train the key parameters and validate the predictive power of the model by using experimental data. The research results indicate that our multicellular system can not only describe the interactions between the microenvironment and cells in detail, but also predict the synergistic effects of drug combinations.

## 1. Introduction

As a complex disease caused by a variety of factors, cancer often involves with multiple gene mutations, signal pathway abnormalities and metabolic changes [1,2]. Since single drug therapy is prone to cause drug resistance, it is currently popular for the scientists and clinicians to explore efficient drug combination therapies from the list of existing drugs [3,4,5], which not only can increase treatment efficacy and reduce toxicity, but also produce a better therapeutic outcome than a single drug under the same application dose condition [6,7]. Therefore, there is potential to treat cancer by studying the synergistic effects of drug combination therapies [8,9].

With the development of information technology, cancer researchers started employing mathematical models to investigate the synergistic effects of drugs [10,11]. The research data mostly come from widely used biotechnology tools, such as protein chips [12], drug-targeting networks [13], molecular pharmacology [14], systems biology approaches [15], genetic interaction networks [16], high-throughput screening [17] and drug transcriptomic profiles [18]. For example, Sun et al. [19] employ the anticancer drug combination target features and cancer gene expression profiles to develop a highly efficient ranking system (RACS) for synergistic anticancer drug combinations. However, the RACS model neither consider the dose effects nor the interactions between cells and the microenvironment in a multicellular system. Then, Sun et al. [20] build up an ordinary differential equations (ODE)-based bone growth model to simulate the dose effects of multiple growth factors, but it still does not describe the multicellular system in detail and lacks an effective specification to validate the predictive power of the model. Moreover, Qiao et al. [21] develop a multi-scale agent- based model to simulate the growth of bone cells under the stimulation of the various drugs, but it does not integrate experimental data to optimize the key parameters of the model.

To overcome the previous shortcomings, this research paper puts forward the following three innovations: (1) it can explore the synergistic effects of drug combinations in a huge dose combinational space at the cell line level; (2) it can simulate the interactions between cells and the microenvironment; (3) it employs both local and global optimization algorithms to train the key parameters and validate the predictive power of the model by integrating the experimental data.

In general, this research develops a predictive model by employing limited experimental data that can simulate and predict not only the effects of a single drug, but also the synergistic effects of drug combinations. Since it considers the cells’ interaction with the microenvironment in a multicellular system, this model can more accurately simulate and predict the synergistic effects of drug combinations than previous research methods [20].

## 2. Materials and Methods

**Figure 1:**
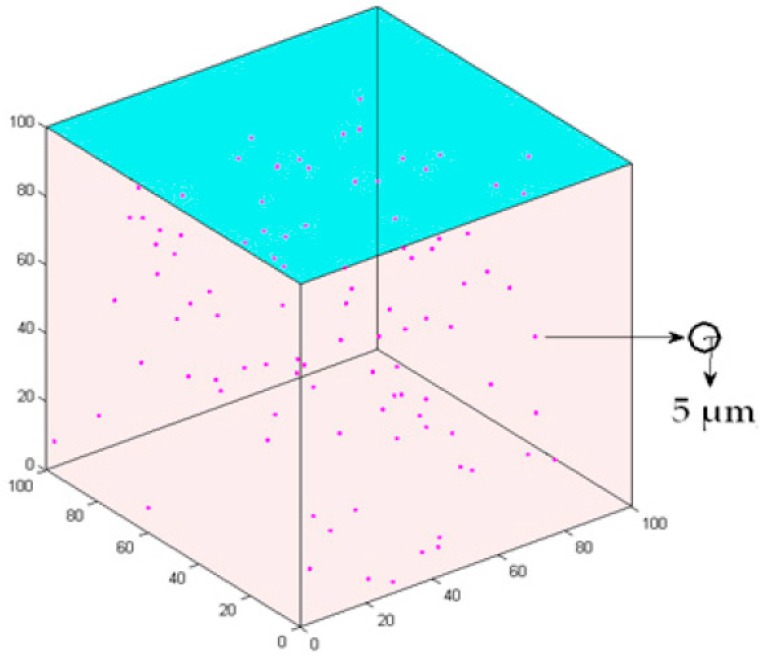
3D cell growth model. Axes x, y and z represent the length, width and height, respectively. Each sphere represents a cell.

This study develops a 3D multiscale agent-based model to simulate the intercellular cell’s competition and the interaction between cancer cells and microenvironment. A 100 × 100 × 100 3D cube according to the previous setup [21,22] is employed to represent the extracellular matrix (ECM) for the microenvironment (Figure 1). The lattice interval is 5 µm, which is approximately the same as the radius of a cancer cell [21]. One hundred cells are initialized in the center of the lattice and the age of the cells is randomly set between 0 and 24 h.

### 2.1. Data Preprocessing

As in our previous study [19], the A549 NSCLC cell line is obtained from the Shanghai Cell Bank (Chinese Academy of Sciences, Shanghai, China). Cells are grown in F12K media supplemented with 10% fetal serum and 100 μg/mL penicillin and 100 μg/mL streptomycin at 37 °C under 5% CO_2_. Drugs are added the day after seeding. The plates are continuously cultured for another 48 h.

Regarding to the cost of the experiment, the biological experiments are only repeated three times for each condition. Limited by the training data size, we have to employ a standard bootstrap method [23,24] to estimate the population means by Equation (1):

(1) $$\bar{X}=\frac{1}{B}\sum_{i=1}^{B}x_{\left(i\right)}$$

Here, $\bar{X}$ represents the estimate mean of the inhibition rate listed by Supplementary Tables S1–S3, *B* is the number of selected samples, *x*_(*i*)_ represents the *i-*th bootstrap sample [25].

### 2.2. Tissue Scale

The main factor affecting the cell microenvironment is the drug concentration at the tissue scale. The half-life [26] is used to describe the change in drug concentration over time. Equation (2) describes the drug dynamics in the 3D extracellular matrix:

(2) $${\mathrm{drug}}_{i}^{t}={\mathrm{drug}}_{i}^{t_{0}}\times {\left(\frac{1}{2}\right)}^{\frac{t}{T}}$$

Here, ${\mathrm{drug}}_{i}^{t_{0}}$ and ${\mathrm{drug}}_{i}^{t}$ are the initial concentration and concentration of the drug at different time point, respectively. *T*, *t*_0_, *t* and *i* represent the half-life, initial time, reaction time and the number of drugs, respectively.

### 2.3. Intracellular Scale: Cell’s Phenotype Switch

**Figure 2:**
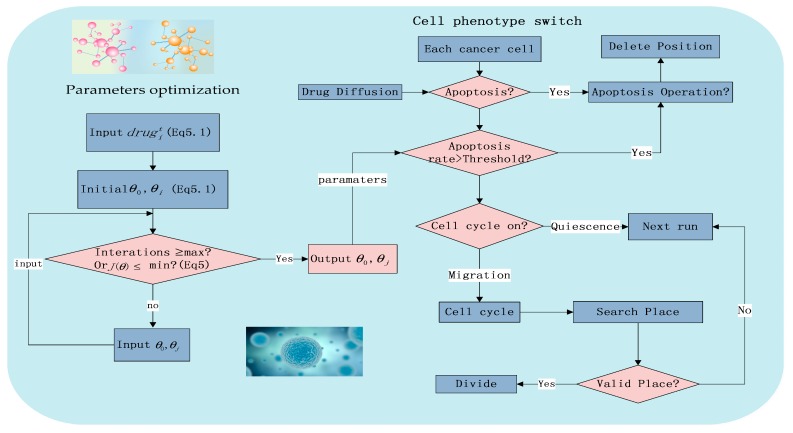
The flowchart of the system including phenotype switching and local parameter optimization.

Due to the previous research [21] and experimental conditions, Figure 2 shows the major workflow in a 72 h experiment.

#### 2.3.1. Apoptosis

##### Natural Mortality Rate of Cancer Cells

According to our previous research [21], Equation (3) [27] is developed to describe the natural mortality rate of cancer cells (*Mnrate*).

(3) $$Mnrate=\left\{ \begin{array}{ll} 1-e^{-\lambda t}, & t>0\\ 0, & t\leq 0 \end{array}\right.$$

*λ* and *t* are positive numbers representing the average apoptosis frequency for each cell and the simulation time, respectively. It should be noted that at each time step, if the apoptosis probability of the cells is less than the threshold, cells will start apoptosis. It will take 10 time steps for cell to complete apoptosis and then be absorbed.

##### Cell’s Mortality Rate and Simulated Cell’s Mortality Rate

Since the experimentalists commonly employ absorbance of the cells [28] to estimate a cell’s mortality rate, this study uses the experimental inhibition rates of the drugs listed in Supplementary Tables S1–S3 to represent cell’s mortality rate (*Merate*) denoted by Equation (4) and the simulated cell’s mortality rate (*Msrate*) is defined by Equation (5):

(4) $$Merate=(1-\frac{Avg_{drug}}{CAvg_{drug}})\times 100\%$$

(5) $$Msrate=\frac{N_{t0}-N_{t1}}{N_{t0}}\times 100\%$$

*Avg_drug_*, *CAvg_drug_*, *N_t_*_0_ and *N_t_*_1_ denote the average absorbance of the drug group, average absorbance of the control group, the number of simulated initial cells and the number of simulated remaining cells, respectively.

##### Parameter Estimation

Equations (6) and (7) are employed to optimize the key parameters of the drug model:

(6) $$Mcrate=\theta_{0}+\theta_{i}\cdot {\mathrm{drug}}_{i}^{t}$$

*Mcrate* is the computed cell’s mortality rate, *θ_i_* (*i* = 0,1,2) are the key parameters of the Equation (6),

(7) $$J(\theta )=\frac{1}{2m}\sum_{j=1}^{m}(Mcrate_{j}-Merate_{j}{)}^{2}$$

*m* represents the number of training sets. *j* is the *i-*th element in the experimental set (Supplementary Tables S1–S3). *J*(*θ*) is the gradient descent method, a loss function to estimate the sum of square about the different between the estimated valve and the true value (Figure 2).

Equation (8) is developed to determine if the cancer cell goes to the apoptosis phenotype or not:

(8) $$c1*Mnrate+c2*Mcrate>P_{rand}$$

where *P_rand_* is a randomly generated number followed by standard uniform distribution [29], the value of which is between 0 and 1. As the left side of Equation (8) is greater than the right side, the cell starts apoptosis and then is absorbed in ten time steps. Here, *c*_1_, *c*_2_ are unknown key parameters of Equation (8).

#### 2.3.2. Proliferation

Equation (9) determines whether the cell enters the cell cycle:

(9) $$\left\{ \begin{array}{l} C_{rand}\in [0,P_{pro})\;\;\;\mathrm{cell}\;\mathrm{cycle}\;\mathrm{ON}\\ C_{rand}\in (P_{pro},1]\;\;\;\mathrm{cell}\;\mathrm{cycle}\;\mathrm{OFF} \end{array}\right.$$

Regarding our previous research [21], in each step we randomly generate a number *C_rand_*. If it falls in the interval [0,Ppro], the cell enters the cell cycle and starts to proliferate. Otherwise, the cell will be in a quiescent state and wait for the next round. If the cancer cells pass through a cell division phase (G0/G1, S and G2) and enter the end of cell division (M phase), a cell will divide if it find at least one free location within the search distance.

#### 2.3.3. Migration

Regarding our previous research [21], non-proliferating cells will migrate at each step. Proliferating cells will migrate in the first three phrases of the cell cycle (G0/G1, S and G2), whereas they will look for an empty location to divide after entering the M phase. This will be discussed in detail in the next section.

#### 2.3.4. Quiescence

Regarding our previous research [21], there are two possibilities for the cell to be in a quiescent state. One is that cell does not enter the cell cycle, and the other is the cell cannot find an appropriate free location for division. If the cell enters the quiescent state, it will wait for a free position to split.

### 2.4. Intercellular Scale

Regarding our previous research [21], cells will select a free position to proliferate or move according to the following rules:

#### 2.4.1. Rule 1

A non-M phase cell at position *P*_0_, it will search for an appropriate position from the six candidate locations of *P*_0_. All candidate positions are ranked by Equations (10)–(12):

(10) $$R_{l}=\frac{1}{4}P(r_{l})\cdot V_{l}$$

(11) $$P(r_{l})=\frac{1}{4\pi D\Delta t}\cdot \exp(\frac{-r_{l}{}^{2}}{4\pi D\Delta t})$$

(12) $$V_{l}=\left\{ \begin{array}{ll} \frac{1}{8}, & P_{ijk}\;\;\text{has 5–6 neighbor cells}\\ \frac{1}{4}, & P_{ijk}\;\;\text{has 3–4 neighbor cells}\\ 1, & P_{ijk}\;\;\text{has 1–2 neighbor cells}\\ \frac{1}{16}, & P_{ijk}\;\;\text{has no neighbor cells} \end{array}\right.$$

where *R_l_* is the ranking score of each candidate position. *r_l_* is the distance from the candidate location *P_ijk_* to position *P*_0_, and *P*(*r_l_*) is the probability that the cell moves to the candidate location *P_ijk_*. *V_l_* is the preference for cell movement.

All the ranks of the candidates are normalized in Equation (13). The sum of all the normalization levels is 1:

(13) $${\tilde{R}}_{l}=\frac{R_{l}}{\sum_{l}R_{l}}$$

All the normalized ranks are incorporated in a *S* set in Equation (14), in which each candidate corresponds to a range *S_l_*:

(14) $$S=\{S_{l}:S_{l}=[\sum \begin{array}{l} m=(l-1)\\ m=0 \end{array}{\tilde{R}}_{m},\tilde{R}+\sum \begin{array}{l} m=(l-1)\\ m=0 \end{array}{\tilde{R}}_{m}]\}$$

Here *S* is an ordered set of *S_l_* .Each *S_l_* is a region in the interval [0,1] and relates to the *i*-th candidate site. If *d* falls in *S_l_*, the corresponding candidate location ${\tilde{R}}_{l}$ will be chosen as the next migration or proliferation site.

#### 2.4.2. Rule 2

If no space is available, the cell will become reversibly quiescent and will wait till the next round.

## 3. Results

This 3D model is implemented in Matlab (R2014a(8.3.0.532),The MathWorks, Inc., Natick, MA, USA), which not only can describe the interactions between cancer cells and the microenvironment under the stimulation of the drugs, but also can predict the synergistic effects of the combination therapy. Moreover, the key parameters and the predictive power of the model are trained and tested by the experimental data listed in Supplementary Tables S1–S3. Table 1 lists the important parameter values of the study.

### 3.1. Local Optimization Results

We optimize the key parameters (*θ_i_* (*i* = 0, 1, 2) of the model (Equation (4)) by the gradient descent method [36] (Figure 2). Table 2 shows the initial estimation value of the key parameters and the small *p* values imply that the optimization result is good enough. Each set of parameters represents a two-drug combinations under 30 different concentration combinations.

### 3.2. Global Optimization Results

Based on the results of Table 2 and Supplementary Tables S1–S3, we use the PSO algorithm [37] and bootstrap algorithm [23,24] to train and validate the key parameters of the model, such as *λ*, *c*_1_, *c*_2_ in Equations (3) and (8).

The experimental data consists 3 combinations of drugs, each consisting of 30 sets of concentrations (Supplementary Tables S1–S3). Here, leave-one-out cross-validation (LOOCV) [38] is employed to validate the predictive power of the model, which uses one third of the experimental data as the training data set to optimize the key parameters and the rest of the data as the testing data set to compute the relative error(RE) for the model precision by Equation (15):

(15) $$RE=\frac{Msrate_{j}(\Theta )-Merate_{j}}{Merate_{j}}$$

Here, Θ is the parameter vector (*c*_1_, *c*_2_, *λ*). Table 3 lists the optimized key parameters of the model. Each set of parameters represents a combination of two different drugs in 30 different concentration combinations.

### 3.3. Spatial Information

**Figure 3:**
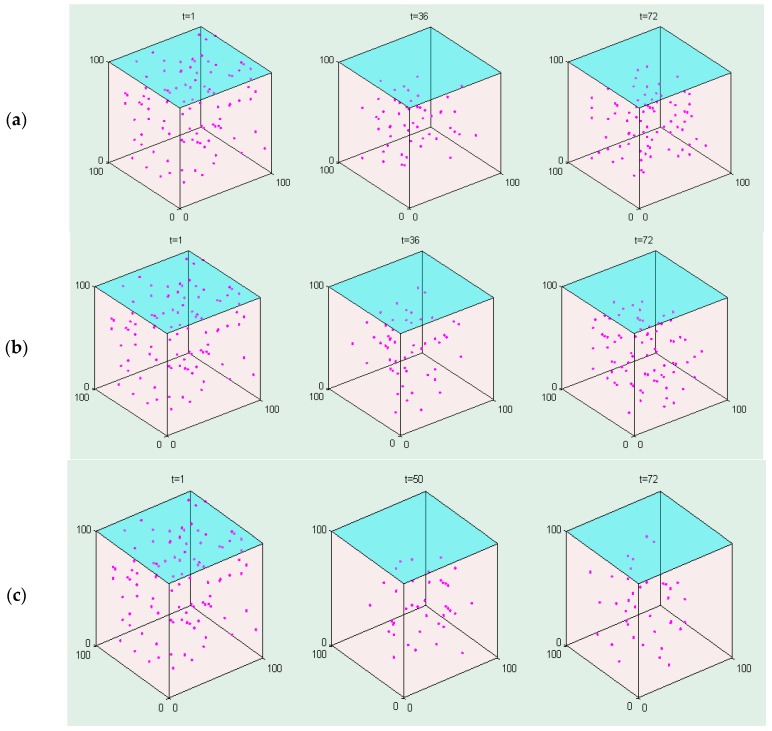
The spatial information of cancer cells under the impact of the (**a**) gefitinib and rosiglitazone; (**b**) erlotinib and imatinib; (**c**) gefitinib and quinacrine. The initial drug ratios between these drug pairs are 1:4.

**Figure 4:**
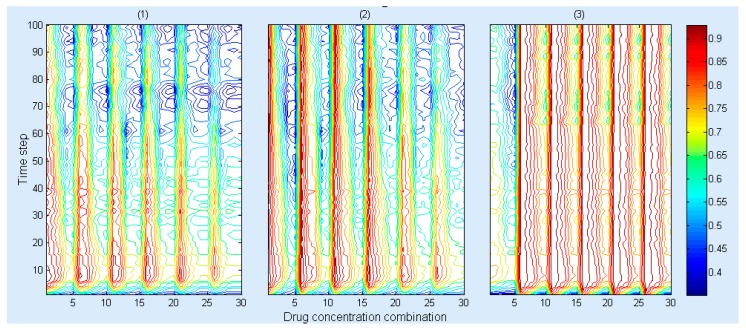
The cell’s mortality rate (*Msrate*) under the different drug concentration combinations for (**1**) gefitinib and rosiglitazone; (**2**) erlotinib and imatinib and (**3**) gefitinib and quinacrine. The x, y axes represent drug concentration combination and time step, respectively, wherein, 10, 20 and 30 on the x axis represent a drug concentration combination detailed in Supplementary Tables S1–S3, respectively. The legend represents the value of the *Msrate*.

This model can show the spatial information of the cancer cells at different times under the same initial drug combination conditions. Figure 3 demonstrates that the number of cancer cells decreases under three drug combinations between 0 and 36 h, and then the number of cancer cells increases after 36 h since the both drug concentration decrease and become less than the initial value (20 μM and 80 μM). Figure 4 shows that the *Msrate* increases at the beginning and then gradually decreases under the same concentration ratio of the drug combinations, whereas a greater concentration ratio of the drug combinations will result in a higher *Msrate* at the same timepoint.

### 3.4. Model Validation

Parameter estimation can be formulated as an optimization problem with the values of state variables as the output of the system (Equation (16)):

(16) $$\Theta^{*}=\underset{\Theta }{\arg\min}\sum_{j=1}^{m}\omega_{j}{\left(Msrate_{j}^{}(\Theta )-Merate_{j}^{}\right)}^{2}$$

where *ω_j_* = (1/max*Merate_j_*)^2^, and *m* denotes the number of drug concentration combination.

**Figure 5:**
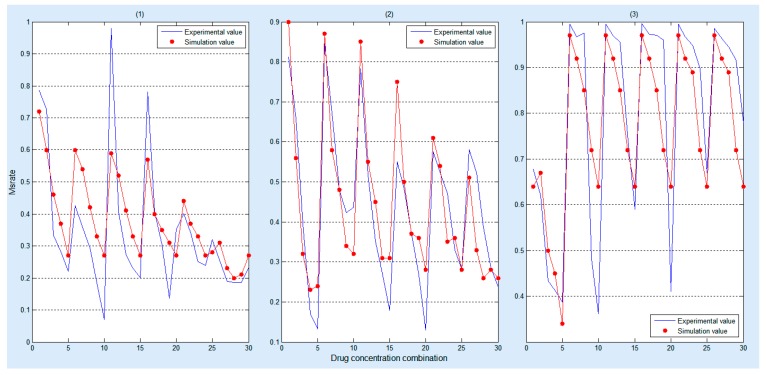
Comparison between simulation and experimental results under the impact of the (**1**) gefitinib and rosiglitazone; (**2**) erlotinib and imatinib; (**3**) gefitinib and quinacrine. The red dots represent the simulation result, and the blue polylines represent the biological experimental data, wherein, 0 to 30 on the X axis represent a drug concentration combination detailed in Supplementary Tables S1–S3, respectively.

Figure 5 demonstrates that our simulation results can approximate the experimental results after training the key parameters of the model by using three different combinations of drugs and each combination has 30 sets of biological experimental data.

Moreover, statistical test [39] is employed to validate the significant difference between the simulated data and the experimental data. The statistical test results are listed in Table 4. Since all the *p*-values are all greater than 0.1, there is no significant difference between the experimental and the simulated results.

### 3.5. The prediction of Synergy Index (CI)

The Loewe Additivity [40] model is a widely accepted method for evaluating synergy with drug combinations, as stipulated in Equation (17) [21]:

(17) $$CI=\frac{d_{1}}{D_{X,1}}+\frac{d_{2}}{D_{X,2}}$$

Here *D_X_*_,1_ and *D_X_*_,2_ represent the single-agent dose or concentration which we input to our model with respect to *X* percentage, respectively. *d*_1_ and *d*_2_ are the combination dose or concentration which we input to our model with respect to *X* percentage. Here, the value of *X* is 50, which represents the fact the *Msrate* is 50 percent. Generally, the effect of a drug combination is described as antagonism if *CI* ≥ 1.1, an additive effect if 0.9 < *CI* < 1.1, synergism if 0.3 ≤ *CI* < 0.9 and strong synergism if *CI* < 0.3 [41].

Drug1 and Drug2 represent the name of a drug, respectively. The CI value on the right indicates the synergistic index obtained for four different concentrations. Since most of the CI simulation result values are close to the experimental results (Table 5), it confirms our CI value predictive capacity. Table 5 shows: (1) when gefitnib and rosigliitazone are combined, we observe an antagonism effect for ratios of 4:1, 3:2, 2:3 and 1:4; (2) when erlotinib and imatinib are combined, we see an additive effect for a 2:3 ratio, while and rest of the ratios (4:1, 3:2 and 1:4) are synergistic; (3) when gefitinib and quinacrine are combined, we observe strong synergism effect for a 3:2 ratio and the rest of the ratios (4:1, 2:3, 1:4) are synergistic.

## 4. Discussion

This study develops a multiscale agent-based model to simulate the synergistic effects of drug combinations in a multicellular system by considering the interaction between cells and the microenvironment in the continuous dose spaces according to the limited experimental data.

As indicated by Table 5, the effects of combinations of gefitinib and rosiglitazone are consistent under four drug combination ratios. So are the effects of the combinations of gefitinib and quinacrine. However, it shows an additive effect for a 2:3 ratio and the synergism for the rest of the ratios (4:1, 3:2 and 1:4) for imatinib and erlotinib combination. According to previous studies, imatinib can cause tumor cell death by targeting Abl and the kit, PDGF and CSF1 receptors [42]. Erlotinib has activity against epidermal growth factor receptor (EGFR) with the function of inhibiting tumor proliferation and differentiation [43]. However, when drugs are used in combination, the effects are highly context- dependent according to previous reports [19]. Generally, such drug combinations are not applicable in clinical use because of their narrow synergy windows. Therefore, more complex and advanced models are needed for these drug combinations with complex concentration ratio-effects.

Compared to the RACS model developed by Sun et al. [19], this model not only can visualize the spatial information of the dynamics of the cancer cell in a 3D microenvironment (Figure 3), but also reveal the dynamic impact of the concentrations of the drug combination on cells’ death rates (Figure 4). Moreover, it employs experimental data (Supplementary Tables S1–S3) to train and validate the key parameters and predictive power of the model (Figure 5), respectively. The research results demonstrate that it can effectively predict the synergistic effects of drug combinations in a complicated multicellular system (Table 5).

However, since the current study is a pilot investigation of the synergistic effects of drug combinations, it has several shortcomings. For example, it does not consider factors like the signaling pathway, drug toxicity and gene mutation. Besides, the dose range of drugs are only limited to 0–100 μM, and the types of drugs are no more than two. Therefore, our further research will integrate more related information and advanced bioinformatics algorithms [44,45,46], to explore the synergistic effects of multiple drugs more accurately in the near future. Next, we will try several state-of-art methods, such as convolutional neural networks [47], deep residual learning [48] and ensemble learning [49] to increase the accuracy of the parameter estimation.

## 5. Conclusions

The research results demonstrate that the model can: (1) train the key parameters and validate the predictive power of the model by using experimental training and testing data; (2) describe a multicellular system in detail by an agent-based model; (3) simulate and predict the synergistic effects of the drug combinations.

## Figures and Tables

**Table 1:** System identified parameters.

Name	Explanation	Value
The half-time of gefitinib	The time it takes to decrease by one half from its initial concentration	48 h [30]
The half-time of imatinib	The time it takes to decrease by one half from its initial concentration	18 h [31]
The half-time of rosiglitazone	The time it takes to decrease by one half from its initial concentration	3~4 h [32]
The half-time of quinacrine	The time it takes to decrease by one half from its initial concentration	5~14 days [33]
The half-time of erlotinib	The time it takes to decrease by one half from its initial concentration	36 h [34]
*P_pro_*	The proliferation rate of cell	0.8 [35]
Cell cycle	The time begin with a completion of a split to the end of the next split	24 time steps
Time step	The time of each simulation step	0.72 h

**Table 2:** Local optimization results.

Drug Combinations	*θ*_0_	*θ*_1_	*θ*_2_	*p*-Value
Gefitinib and rosiglitazone	0.1686	0.0067	0.0019	0.0137
Erlotinib and imatinib	0.1789	0.0076	0.0031	0.0083
Gefitinib and quinacrine	0.0006	0.0085	0.0101	0.0374

**Table 3:** The parameters value obtained by PSO.

Drug Combinations	*c*_2_	*c*_1_	*λ*	RE of Equation (15)
Gefitinib and rosiglitazone	0.218	0.124	1	0.0636
Erlotinib and imatinib	0.3704	0.0081	1	0.2357
Gefitinib and quinacrine	0.4722	0.2303	1	0.8331

**Table 4:** The significance test results.

Significance Test	*p*-Value
Gefitinib and rosiglitazone	0.1955
Erlotinib and imatinib	0.9470
Gefitinib and quinacrine	0.1331

**Table 5:** The comparison of CI values.

Drug1	Drug2	Data Types	CI Value			
			4:1	3:2	2:3	1:4
Gefitinib	Rosiglitazone	Experimental data	[1.34,1.66]	[1.65,1.93]	[1.83,1.91]	[2.4,2.52]
		Simulation data	1.219	1.6008	1.3785	1.1255
Erlotinib	Imatinib	Experimental data	[0.42,0.44]	[0.71,1.01]	[1.06,1.54]	[0.67,1.17]
		Simulation data	0.7269	0.8002	1.0694	0.8665
Gefitinib	Quinacrine	Experimental data	[0.54,0.72]	[0.22,0.34]	[0.5,0.58]	[0.3,0.46]
		Simulation data	0.303	0.26	0.3606	0.821

## Supplementary Materials

The supplementary materials are available online.

## References

[1] Hill J.A., Cowen L.E. (2015). Using combination therapy to thwart drug resistance. Future Microbiol..

[2] Nanasinkam S.P., Powell C.A. (2013). Molecular Biology of Lung Cancer. J. Thorac. Dis..

[3] Kayakiri H., Kato T., Minoura H., Hirosumi J. (2005). Concomitant Drugs. U.S. Patent.

[4] Andronis C., Sharma A., Deftereos S., Virvilis V., Konstanti O., Persidis A., Persidis A., Barratt M.J., Frail D.E. (2012). Mining scientific and clinical databases to identify novel uses for existing drugs. Drug Repositioning: Bringing New Life to Shelved Assets and Existing Drugs.

[5] Borges R. (2014). We need a global system to help identify new uses for existing drugs. BMJ.

[6] Allazikani B., Banerji U., Workman P. (2012). Combinatorial drug therapy for cancer in the post-genomic era. Nat. Biotechnol..

[7] Möttönen T., Hannonen P., Leirisalo-Repo M., Nissilä M., Kautiainen H., Korpela M., Laasonen L., Julkunen H., Luukkainen R., Vuori K. (1999). Comparison of combination therapy with single-drug therapy in early rheumatoid arthritis: A randomised trial. Lancet.

[8] Versi E. (2003). Combination Therapy. U.S. Patent.

[9] Yin N., Ma W., Pei J., Ouyang Q., Tang C., Lai L. (2014). Synergistic and Antagonistic Drug Combinations Depend on Network Topology. PLoS ONE.

[10] Ryall K.A., Tan A.C. (2015). Systems biology approaches for advancing the discovery of effective drug combinations. J. Cheminform..

[11] Davidov E., Holland J., Marple E., Naylor S. (2003). Advancing drug discovery through systems biology. Drug Discov. Today.

[12] Feng Y., Wang B., Chu X., Wang Y., Zhu L. (2016). The Development of Protein Chips for High Throughput Screening (HTS) of Chemically Labeling Small Molecular Drugs. Mini Rev. Med. Chem..

[13] Huang L., Li F., Sheng J., Xia X., Ma J., Zhan M., Wong S.T.C. (2014). DrugComboRanker: Drug combination discovery based on target network analysis. Bioinformatics.

[14] Zhao X.M., Iskar M., Zeller G., Kuhn M., Van N.V., Bork P. (2011). Prediction of Drug Combinations by Integrating Molecular and Pharmacological Data. PLoS Comput. Biol..

[15] Wu Z., Zhao X.M., Chen L. (2010). A systems biology approach to identify effective cocktail drugs. BMC Syst. Biol..

[16] Wang Y.Y., Xu K.J., Song J., Zhao X.M. (2012). Exploring drug combinations in genetic interaction network. BMC Bioinform..

[17] Eldridge G.R., Vervoort H.C., Lee C.M., Cremin P.A., Williams C.T., Hart S.M., Goering M.G., O’Neiljohnson M., Zeng L. (2002). High-throughput method for the production and analysis of large natural product libraries for drug discovery. Anal. Chem..

[18] Sirota M., Dudley J.T., Kim J., Chiang A.P., Morgan A.A., Sweetcordero A., Sage J., Butte A.J. (2011). Discovery and preclinical validation of drug indications using compendia of public gene expression data. Sci. Transl. Med..

[19] Sun Y., Sheng Z., Ma C., Tang K., Zhu R., Wu Z., Shen R., Feng J., Wu D., Huang D. (2015). Combining genomic and network characteristics for extended capability in predicting synergistic drugs for cancer. Nat. Commun..

[20] Sun X., Su J., Bao J., Peng T., Zhang L., Zhang Y., Yang Y., Zhou X. (2012). Cytokine combination therapy prediction for bone remodeling in tissue engineering based on the intracellular signaling pathway. Biomaterials.

[21] Qiao M., Wu D., Carey M., Zhou X., Zhang L. (2015). Multi-Scale Agent-Based Multiple Myeloma Cancer Modeling and the Related Study of the Balance between Osteoclasts and Osteoblasts. PLoS ONE.

[22] Wang J., Le Z., Jing C., Gang Y., Wu H., Miao H., Wu Y., Zhou X. (2013). Multi-scale agent-based modeling on melanoma and its related angiogenesis analysis. Theor. Biol. Med. Model..

[23] Davison A.C., Hinkley D.V. (2013). Bootstrap Methods and Their Application.

[24] MacKinnon J.G. (2006). Bootstrap Methods in Econometrics. Econ. Rec..

[25] Arcones M.A., Giné E. (1989). The bootstrap of the mean with arbitrary bootstrap sample size. Annales de l’I. H. P. Section B.

[26] Clairambault J. (2013). Half-life Time. Encycl. Syst. Biol..

[27] Goldie C.M., Klüppelberg A. (2010). Subexponential distributions. Reliab. Eng. Syst. Saf..

[28] Lu C. (1998). Apply a MTT assay to studying on anti-HIV drugs. China J. Basic Med. Tradit. Chin. Med..

[29] Hasan O. (2007). Standard Uniform Distribution Theory in HOL4.

[30] Ranson M., Wardell S. (2004). Gefitinib, a novel, orally administered agent for the treatment of cancer. J. Clin. Pharm. Ther..

[31] Pavlovsky C., Egorin M.J., Shah D.D., Beumer J.H., Rogel S., Pavlovsky S. (2009). Imatinib Mesylate Pharmacokinetics Before and After Sleeve Gastrectomy in a Morbidly Obese Patient with Chronic Myeloid Leukemia. Pharmacotherapy.

[32] Sheu W.H., Chuang H.C., Cheng S.M., Lee M.R., Chou C.C., Cheng F.C. (2011). Microdialysis combined blood sampling technique for the determination of rosiglitazone and glucose in brain and blood of gerbils subjected to cerebral ischemia. J. Pharm. Biomed. Anal..

[33] USP Convention. U.S.P. (1997). Quinacrine Systemic.

[34] Petit-Jean E., Buclin T., Guidi M., Quoix E., Gourieux B., Decosterd L.A., Gairard-Dory A.C., Ubeaud-Séquier G., Widmer N. (2015). Erlotinib: Another candidate for the therapeutic drug monitoring of targeted therapy of cancer? A pharmacokinetic and pharmacodynamic systematic review of literature. Ther. Drug Monit..

[35] Kastan M.B., Bartek J. (2004). Cell-cycle checkpoints and cancer. Nature.

[36] Kumar D., Gupta S., Sehgal P. Comparing gradient based learning methods for optimizing predictive neural networks. Proceedings of the 2014 Recent Advances in Engineering and Computational Sciences.

[37] Zhou C., Gao H.B., Gao L., Zhang W.G. (2003). Particle Swarm Optimization (PSO) Algorithm. Appl. Res. Comput..

[38] Cawley G.C., Talbot N.L.C. (2003). Efficient leave-one-out cross-validation of kernel fisher discriminant classifiers. Pattern Recognit..

[39] Yuan X., Yang C., Nan H., Yang Z., He X., Li T., Le Z. (2017). Exploring the key genes and signaling transduction pathways related to the survival time of glioblastoma multiforme patients by a novel survival analysis model. BMC Genom..

[40] Lee J.J., Kong M., Ayers G.D., Lotan R. (2007). Interaction index and different methods for determining drug interaction in combination therapy. J. Biopharm. Stat..

[41] Suárez-Arroyo I.J., Rios-Fuller T.J., Feliz-Mosquea Y.R., Mercedes L.V., Leal-Alviarez D.J., Gerónimo M.M., Cubano L.A., Martínez-Montemayor M.M. (2016). Ganoderma lucidumCombined with the EGFR Tyrosine Kinase Inhibitor, Erlotinib Synergize to Reduce Inflammatory Breast Cancer Progression. J. Cancer.

[42] Gorzalczany Y., Gilad Y., Amihai D., Hammel I., Sagi-Eisenberg R., Merimsky O. (2011). Combining an EGFR directed tyrosine kinase inhibitor with autophagy-inducing drugs: A beneficial strategy to combat non-small cell lung cancer. Cancer Lett..

[43] Pao W., Chmielecki J. (2010). Rational, biologically based treatment of EGFR-mutant non-small-cell lung cancer. Nat. Rev. Cancer.

[44] Zhang L., Qiao M., Gao H., Hu B., Tan H., Zhou X., Li C.M. (2016). Investigation of mechanism of bone regeneration in a porous biodegradable calcium phosphate (CaP) scaffold by a combination of a multi-scale agent-based model and experimental optimization/validation. Nanoscale.

[45] Zhang L., Zhang S. (2017). Using game theory to investigate the epigenetic control mechanisms of embryo development: Comment on: “Epigenetic game theory: How to compute the epigenetic control of maternal-to-zygotic transition” by Qian Wang et al. Phys. Life Rev..

[46] Zhang L., Zheng C., Li T., Xing L., Zeng H., Li T., Yang H., Cao J., Chen B., Zhou Z. (2017). Building Up a Robust Risk Mathematical Platform to Predict Colorectal Cancer. Complexity.

[47] Kim Y. (2014). Convolutional Neural Networks for Sentence Classification. arXiv.

[48] Ishikawa Y., Washiya K., Aoki K., Nagahashi H. (2016). Brain Tumor Classification of Microscopy Images Using Deep Residual Learning. Proc. SPIE.

[49] Webb G.I., Zheng Z. (2004). Multistrategy Ensemble Learning: Reducing Error by Combining Ensemble Learning Techniques. IEEE Trans. Knowl. Data Eng..

