# Supplementary material for: Developing an Agent-Based Drug Model to Investigate the Synergistic Effects of Drug Combinations
Source: Molecules. 2017 Dec 14;22(12):2209. doi: 10.3390/molecules22122209 (PMC6149923; doi:10.3390/molecules22122209)
Supplement: Supplementary file 1 [file molecules-22-02209-s001.pdf]

molecules

Article

Developing an Agent-Based Drug Model to Investigate the Synergistic Effects of Drug Combinations

Hongjie Gao [1], Zuojing Yin [2], Zhiwei Cao [2,*] and Le Zhang [1,3,*]

[1] College of Computer and Information Science, Southwest University, Chongqing 400715, China; ghjbarry@126.com
[2] School of Life and Technology, Tongji University, Shanghai 200092, China; ZuoJing_Yin@tongji.edu.cn
[3] College of Computer Science, Sichuan University, Chengdu 610065, China
* Correspondence: zwcao@tongji.edu.cn (Z.C.); zhangle06@scu.edu.cn (L.Z.)

Supplementary Table 1: Experimental data for the drugs gefitinib and rosiglitazone.

Concentration ratio	Concentration of gefitinib	Concentration of rosiglitazone	Experimental data 1	Experimental data 2	Experimental data 3	Mean	Inhibition rate
5/0	100	0	0.06	0.048	0.063	0.058	0.7863
	50	0	0.071	0.095	0.068	0.074	0.726
	25	0	0.176	0.187	0.184	0.183	0.332
	12.5	0	0.201	0.193	0.192	0.196	0.282
	6.25	0	0.216	0.213	0.214	0.213	0.22
4/1	80	20	0.148	0.161	0.167	0.157	0.4262
	40	10	0.165	0.187	0.181	0.176	0.3575
	20	5	0.199	0.192	0.189	0.193	0.2935
	10	2.5	0.237	0.222	0.22	0.223	0.185
	5	1.25	0.268	0.25	0.242	0.255	0.07
3/2	60	40	0.004	0.006	0.008	0.005	0.978
	30	20	0.173	0.155	0.158	0.162	0.4063
	15	10	0.207	0.197	0.194	0.199	0.2728
	7.5	5	0.207	0.201	0.232	0.210	0.231
	3.8	2.5	0.219	0.217	0.224	0.219	0.2016
2/3	40	60	0.049	0.062	0.065	0.059	0.7815
	20	30	0.168	0.163	0.156	0.162	0.4066
	10	15	0.195	0.19	0.186	0.191	0.3036
	5	7.5	0.232	0.238	0.242	0.236	0.1362
	2.5	3.8	0.172	0.177	0.182	0.178	0.3505
1/4	20	80	0.158	0.163	0.169	0.164	0.4004
	10	40	0.178	0.184	0.187	0.180	0.34
	5	20	0.211	0.208	0.197	0.205	0.249
	2.5	10	0.219	0.186	0.235	0.208	0.238
	1.25	5	0.188	0.205	0.166	0.186	0.32
0/5	0	100	0.199	0.204	0.209	0.204	0.253
	0	50	0.219	0.223	0.225	0.222	0.189
	0	25	0.227	0.225	0.22	0.223	0.186
	0	12.5	0.219	0.228	0.219	0.223	0.186
	0	6.25	0.217	0.21	0.203	0.210	0.23
Negative Control	0	0	0.219	0.223	0.227	0.223	/

Supplementary Table 2: Experimental data for the drugs erlotinib and imatinib.

Concentration ratio	Concentration of erlotinib	Concentration of imatinib	Experimental data 1	Experimental data 2	Experimental data 3	Mean	Inhibition rate
5/0	100	0	0.111	0.113	0.112	0.1121	0.8118
	50	0	0.211	0.195	0.206	0.2038	0.6579
	25	0	0.351	0.371	0.356	0.3598	0.3959
	12.5	0	0.472	0.508	0.505	0.4937	0.1712
	6.25	0	0.494	0.514	0.549	0.5171	0.1319
4/1	80	20	0.095	0.086	0.096	0.0923	0.8450
	40	10	0.229	0.182	0.196	0.1957	0.6714
	20	5	0.278	0.315	0.326	0.3107	0.4784
	10	2.5	0.299	0.338	0.36	0.3438	0.4227
	5	1.25	0.317	0.348	0.337	0.3358	0.4363
3/2	60	40	0.113	0.141	0.139	0.1293	0.7829
	30	20	0.298	0.275	0.294	0.2879	0.5166
	15	10	0.353	0.401	0.377	0.3862	0.3516
	7.5	5	0.400	0.433	0.445	0.4344	0.2707
	3.8	2.5	0.442	0.504	0.488	0.4895	0.1782
2/3	40	60	0.300	0.257	0.262	0.2681	0.5499
	20	30	0.309	0.289	0.315	0.3073	0.4841
	10	15	0.381	0.363	0.372	0.3741	0.3720
	5	7.5	0.465	0.381	0.432	0.4366	0.2670
	2.5	3.8	0.511	0.514	0.53	0.5188	0.1291
1/4	20	80	0.254	0.251	0.254	0.2528	0.5755
	10	40	0.275	0.28	0.289	0.2837	0.5237
	5	20	0.318	0.301	0.327	0.3148	0.4714
	2.5	10	0.392	0.395	0.407	0.3996	0.3291
	1.25	5	0.418	0.432	0.446	0.4277	0.2820
0/5	0	100	0.241	0.258	0.245	0.2498	0.5807
	0	50	0.288	0.278	0.286	0.2842	0.5229
	0	25	0.401	0.345	0.358	0.3652	0.386
	0	12.5	0.416	0.346	0.425	0.4255	0.2857
	0	6.25	0.456	0.445	0.465	0.4545	0.2369
Negative Control	0	0	0.581	0.572	0.598	0.584	/

Supplementary Table 3: Experimental data for the drugs gefitinib and quinacrine.

Concentration ratio	Concentration of gefitinib	Concentration of quinacrine	Experimental data 1	Experimental data 2	Experimental data 3	Mean	Inhibition rate
5/0	100	0	0.160	0.193	0.1820	0.1777	0.6779
	50	0	0.2350	0.1990	0.2050	0.2100	0.6194
	25	0	0.2970	0.306	0.331	0.3128	0.4332
	12.5	0	0.355	0.3280	0.3110	0.3243	0.4124
	6.25	0	0.373	0.323	0.348	0.3383	0.3869
4/1	80	20	0.004	0.0020	0.0030	0.0030	0.9945
	40	10	0.021	0.0168	0.0177	0.0183	0.9667
	20	5	0.013	0.016	0.011	0.0139	0.9747
	10	2.5	0.278	0.291	0.293	0.2863	0.4813
	5	1.25	0.336	0.382	0.369	0.3522	0.3619
3/2	60	40	0.0020	0.0030	0.0040	0.0031	0.9942
	30	20	0.0180	0.0160	0.017	0.0169	0.9693
	15	10	0.0230	0.029	0.0230	0.0243	0.9558
	7.5	5	0.124	0.143	0.133	0.1362	0.7532
	3.8	2.5	0.2130	0.2260	0.235	0.2261	0.5903
2/3	40	60	0.002	0.0010	0.0030	0.0023	0.9958
	20	30	0.0130	0.0180	0.0140	0.0150	0.9728
	10	15	0.0170	0.0170	0.0140	0.0165	0.97
	5	7.5	0.0190	0.0260	0.0150	0.0221	0.9598
	2.5	3.8	0.3310	0.3210	0.327	0.3251	0.41
1/4	20	80	0.0030	0.0020	0.0040	0.0031	0.994
	10	40	0.0180	0.0150	0.0210	0.0180	0.96
	5	20	0.0260	0.0380	0.0270	0.0283	0.948
	2.5	10	0.0630	0.0490	0.060	0.0562	0.898
	1.25	5	0.1660	0.2040	0.1850	0.1820	0.6701
0/5	0	100	0.0080	0.0060	0.0080	0.0076	0.986
	0	50	0.019	0.021	0.020	0.0200	0.9637
	0	25	0.034	0.029	0.028	0.0302	0.9452
	0	12.5	0.055	0.039	0.0440	0.0463	0.9161
	0	6.25	0.129	0.119	0.1180	0.1223	0.7782
Negative Control	0	0	0.519	0.55	0.588	0.552	/

© 2017 by the authors. Submitted for possible open access publication under the terms and conditions of the Creative Commons Attribution (CC BY) license (http://creativecommons.org/licenses/by/4.0/).
